# Medical Device Integration Model Based on the Internet of Things

**DOI:** 10.2174/1874120701509010256

**Published:** 2015-09-17

**Authors:** Aiyu Hao, Ling Wang

**Affiliations:** 1 Institute of Software Services Outsourcing, Suzhou Institute of Industrial Technology, Suzhou 215000, China; 2 College of Mechanical & Electronic Engineering, Henan Agriculture University, Zhengzhou 450002, China

**Keywords:** Data integration, HIS system, medical devices, medical device integration (MDI)

## Abstract

At present, hospitals in our country have basically established the HIS system, which manages registration, treatment, and charge, among many others, of patients. During treatment, patients need to use medical devices repeatedly to acquire all sorts of inspection data. Currently, the output data of the medical devices are often manually input into information system, which is easy to get wrong or easy to cause mismatches between inspection reports and patients. For some small hospitals of which information construction is still relatively weak, the information generated by the devices is still presented in the form of paper reports. When doctors or patients want to have access to the data at a given time again, they can only look at the paper files. Data integration between medical devices has long been a difficult problem for the medical information system, because the data from medical devices are lack of mandatory unified global standards and have outstanding heterogeneity of devices. In order to protect their own interests, manufacturers use special protocols, etc., thus causing medical decices to still be the "lonely island" of hospital information system. Besides, unfocused application of the data will lead to failure to achieve a reasonable distribution of medical resources. With the deepening of IT construction in hospitals, medical information systems will be bound to develop towards mobile applications, intelligent analysis, and interconnection and interworking, on the premise that there is an effective medical device integration (MDI) technology. To this end, this paper presents a MDI model based on the Internet of Things (IoT). Through abstract classification, this model is able to extract the common characteristics of the devices, resolve the heterogeneous differences between them, and employ a unified protocol to integrate data between devices. And by the IoT technology, it realizes interconnection network of devices and conducts associate matching between the data and the inspected at the device terminal in a timely manner.

## 1. RELEVANT WORK AND TECHNOLOGY

### 1.1 Relevant Work

The difficulties in MDI lie in the data standards between heterogeneous devices as well as data acquisition modes. In order to allow different devices to achieve interconnection and interworking in a uniform data representation manner, there have some international standards such as HL7, DICOM, CDA, IHE, etc. Grounded upon the SOA technology, literature [1] and [2] put forward a data integration scheme. However, the scheme is dependent on the above standards, while these standards are not mandatory and are not underpinned by all manufacturers’ devices. For devices that are not based on standards, data integration remains difficult. Literature [3] presents the form of gateway to integrate medical devices, but similarly, data integration is still dependent on the standards, and the transceiver protocol between the gateway and the medical devices are all complex. Literature [4] only discussed the storage formats of medical device data, while most importantly, it has neglected the exploration of data collection. Literature [5] proposes a model of software dynamic evolution, which has some reference value for such application scenarios with rapidly changing demands like MDI.

### 1.2 Relevant Technology

The IoT technology refers to connect all objects by using the information sensing devices to the Internet for information exchange, which is exchanging of physical objects, in order to achieve intelligent identification and management. In this paper, the IoT technology is employed to identify and manage medical devices and the inspected.

The U.S. National Institute of Standards and Technology (NIST) defines cloud computing as a model for enabling ubiquitous, convenient, on-demand network access to a shared pool of configurable computing resources (e.g., networks, servers, storage, applications, and services) that can be rapidly provisioned and released with minimal management effort or service provider interaction. In this paper, cloud computing serves as a filtering and storage carrier for data integration.

The dynamic evolution of software technology is an effective means to meet the open environment of the Internet and also the ever-changing needs of users. It is also the core technology for autonomic computing, grid computing, adaptive software, and network configuration software.

## 2. MODELING

The MDI model (hereinafter referred to MDIM) based on the IoT technology as presented in this paper is composed of four parts, which are the device abstraction layer, device adaptation layer, data extraction layer, data filtering layer and integration layer.

### 2.1 Medical Device Abstraction Layer

Despite the diversity and heterogeneity of medical devices, focusing on the data integration problem, medical devices can be abstracted and classified by the data characteristics of the devices, and this is what the device abstraction layer does. At this layer, abstract modeling should be first built for the medical devices and the following model can be abstracted as per the data output type of devices:

MD = {standardized output devices, non-standard imaging devices, non-standard value devices}

Wherein, standardized output devices refer to standard devices that support standards such as HL7, DICOM, CDA, and IHE, and to integrate such devices, a number of existing mature schemes can be referred to. For non-standard devices, since they do not support a uniform standard, the data generated by the devices don’t have a fixed format, which causes a great difficulty to integrate. These devices are objects that this model focuses on, and such devices can be subdivided into imaging devices and numerical devices in this model.

### 2.2 Device Adaptation Layer

The device adaptation layer is to resolve the association problem of the device and its functions. For a set of device, its function is to collection of the inspection items that it can manage. There is a 1: n relationship between a device and an inspection item. In this paper, the device functions are used to build the model below:

MDF = {collection of inspection items, device type, communication mode} 

Wherein, the device type is classified into three categories by the MD, while the corresponding communication mode may be any serial or Ethernet port. The MD and MDF are corresponded to complete the adaptation of medical devices.

### 2.3 Data Acquisition Layer

A prerequisite for the integration of medical devices is that medical devices should be accessed to an intercommunicating network before acquiring data from them. The effect of the data extraction layer is firstly to provide networking capabilities to the device, and then submit the device data acquired to the next layer for processing. Meanwhile, a matching problem between the device data and the inspected should be addressed at this layer. Different from other parts, the realization of this layer’s functions should be combined with hardware.

### 2.4 Data Filtering Layer

Data acquired at the acquisition layer may be imaging reports, or formatting output of values. Some are standard while some are custom protocols by manufacturers, and the latter cannot be directly put into storage. It is indispensable to establish uniform rules to filter these data, and this is work at the data filtering layer. At this layer, the model builds a uniform protocol as the basis of data filtering and also the data storage format:

MDIDP = {inspection items, inspection end value, reference value, inspection conclusions}

The numerical results can be directly corresponded to each item in the protocol. For the imaging results, the storage location of imaging files is corresponding to the inspection end value in the protocol.

## 3. REALIZATION OF THE MODEL

### 3.1 Realization of the Device Layer

The device abstraction layer and the device adaptation layer are realized mainly through the OOP technology, which objectifies medical devices. As the definition model of medical devices in Section 2.1, the UML diagram can be applied to show the final realization class diagram.

### 3.2 Realization of the Data Acquisition Layer

The data abstraction layer is achieved mainly by using hardware MCU as a data gateway. For each set of medical device, there is MCU hardware to connect with it. According to the output type of medical devices, two connection types are available: RS232 or two RJ45. The design of MCU is based on the IoT technology. MCU can have access to the LAN or the Internet through wireless means. Each MCU has a unique identification code. Therefore, medical devices gain the networking capabilities by docking MCU, while there is a one-to-one relationship between the device and the MCU. The identification of MCU can be used as the identification of the device in the network, and thus the IoT of medical devices has been set up.

The final inspection results of medical devices will be sent out via RS232 or RJ45. The docking MCU has special listening services. If there is data transmission, MCU will extract the data first and then transmit them to the cloud server. In order to quickly exchange data and reduce the burden on the MCU, MCU is only responsible for data extraction and forwarding, and not responsible for other calculations. MCU extracts data transmitted by the device from the communication layer, so it can be used universally by all of the medical devices. Further, it does not intrude into the medical devices, and thus data output by devices that do not support HL7, DICOM protocols, etc. can also be collected.

Data obtained from medical devices should also be associated with detailed information of patients. For HL7, DICOM and other protocols, the messages themselves will carry patient information. However, it is impossible for non-standard imaging devices and non-standard numerical devices to transmit such data. As a consequence, the output results of these devices will easily lead to mismatches and eventually turn into useless data. By integrating RFID, two-dimensional code recognition, barcode recognition and other functions on the MCU, information of patients inspected can be obtained. Each time the MCU forwards the data to the cloud, it is triggered by recognizing patient information (swiping cards or scanning codes, etc.), thereby forming a two-tuples [pid, data] to be uploaded to the cloud layer and ensuring the data are always linked to the corresponding numbers of patients.

### 3.3 Realization of the Data Filtering Layer

The final data exchange protocol MDIDP of this model is described in Section 2.4. As per the modeling of the device in Section 2.1, the data filtering layer will transform the protocols of the following three types of data:

(1) For standard devices, the data filtering layer will analyze the packets of such protocols as HL7 and DICOM to obtain the desired fields.

(2) For output of non-standard imaging devices, the data filtering layer will take the storage path of the image files as the inspection end value.

(3) The output of non-standard value devices does not have a standard and unified data format. Each manufacturer has their definition of the output data. Confronting with such a changing and heterogeneous scene, the data filtering layer is realized through the dynamic evolution of software technology. First of all, the data standards of these manufacturers are expressed regularly. Afterwards, by reflection, regular matching is conducted on these data in the implementation phrase and eventually the corresponding data are obtained.

## 4. EVOLUTION AND EXPANSION OF THE MODEL

When applying this model, the integration of each set of medical device poses a new demand. Devices are from different manufacturers and have different data formats and standards, as well as different types of output. For the model, demand is constantly evolving, which is a typical demand evolution scenario. Through hierarchical design, this model has the ability of dynamically evolving. When there is adapting demand for new devices, it is fine just to add the device in the background, complete device adaptation well and establish the rules for data filtering. There is no need to change the system implementation codes, and the data filtering layer can filter the data as per the configured rules at runtime.

In this model, the operation carriers are cloud computing infrastructure, server, database, and storage, which are all provided by cloud service providers. The cloud services undertake the data filtering tasks of the model, and the end MCU is only in charge of forwarding the underlying data to the cloud, so as to save bandwidth and computing resources, as well as dramatically lowering maintenance costs of the entire system. As long as in the interconnection state, data provided by the system are available. A unified data storage format also provides convenience to the back-end extensions of the model. Whether it is pc terminal, mobile app terminal or WeChat public account, all have access to the system data. The data are pushed to the nearest terminal of the users, which breaks the visit barrier. Different hospitals and doctors can easily exchange data, and patients’ data can be saved permanently. Adding some video technologies can achieve remote mobile medical care.

## 5. CASE STUDY

This model research comes from the school-enterprise cooperation project. At present it realizes this model in joint collaboration with Suzhou Zhaocheng Software Co., Ltd. Software systems grounded on this model have been tried out in a number of hospitals, and the background has collected the practical data from multiple types of devices, which verify the correctness and validity of this model.

## CONCLUSION

Mobile healthcare and telemedicine of the Internet thinking are new development directions for the healthcare information system. The premise to achieve this goal is that data of medical devices can be interconnected with the Internet and acquired at anytime and anywhere. Currently, the hospital information system still cannot achieve it. The MDI model based on the IoT technology in this paper can be compatible with the vast majority of medical devices on the market, achieve seamless integration of data, and resolve the data sharing problem of medical devices. Based on the IoT and cloud computing platform, this model allows information to interconnect and interwork at anytime and anywhere, thus laying a solid foundation for mobile healthcare and telemedicine and presenting some market value.

## Figures and Tables

**Fig. (1):**
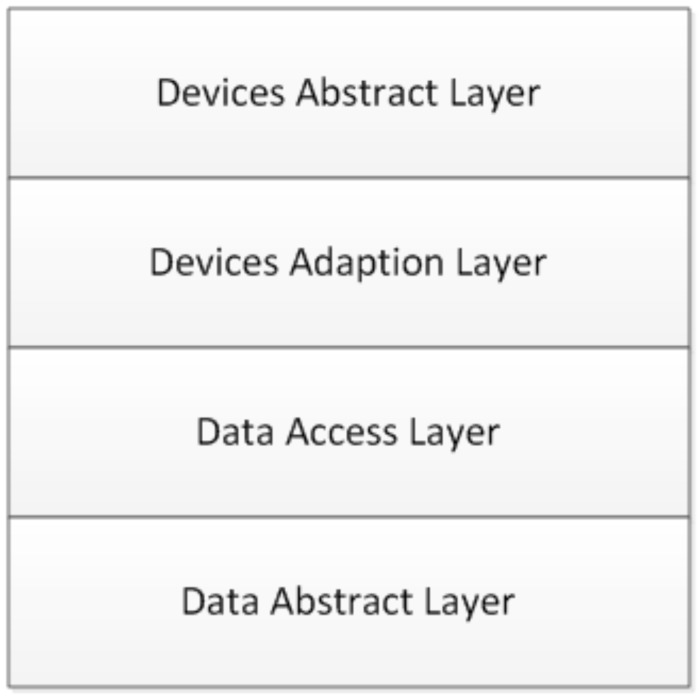
Model structure diagram.

**Fig. (2):**
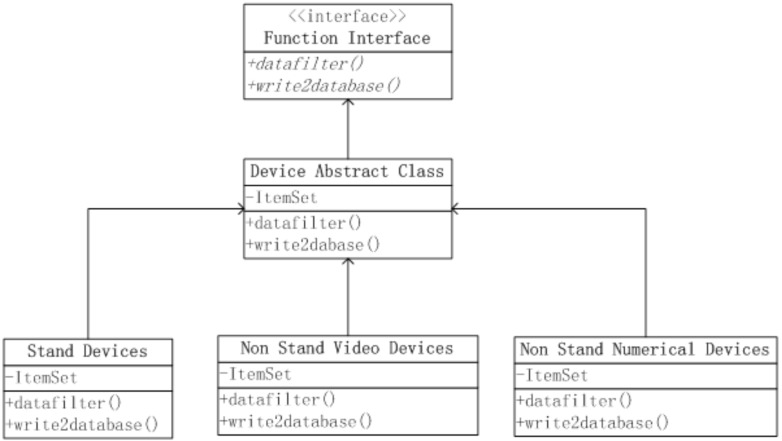
UML diagram at the device layer.

**Fig. (3):**
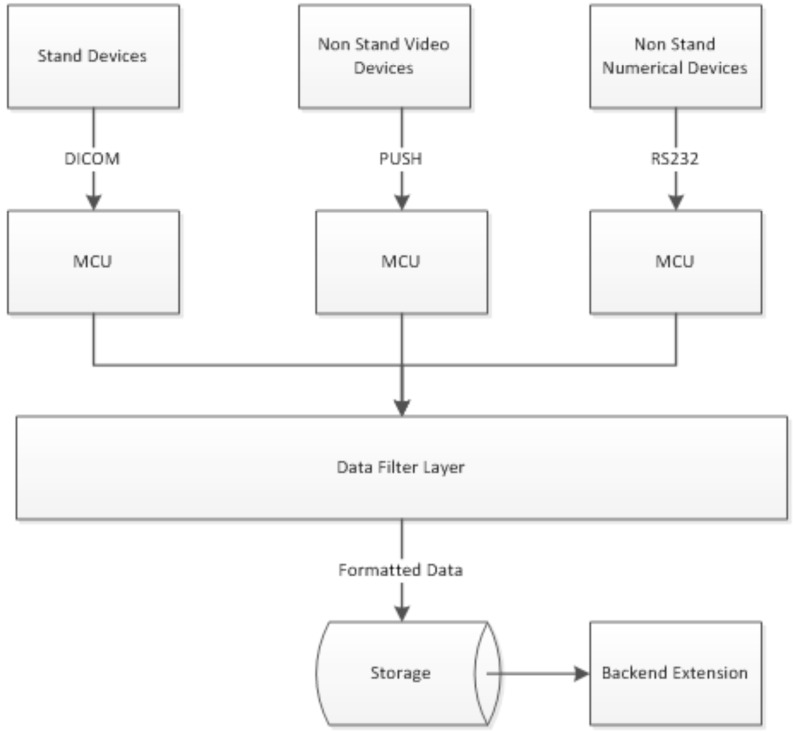
Structure map of the data acquisition layer.

**Fig. (4):**
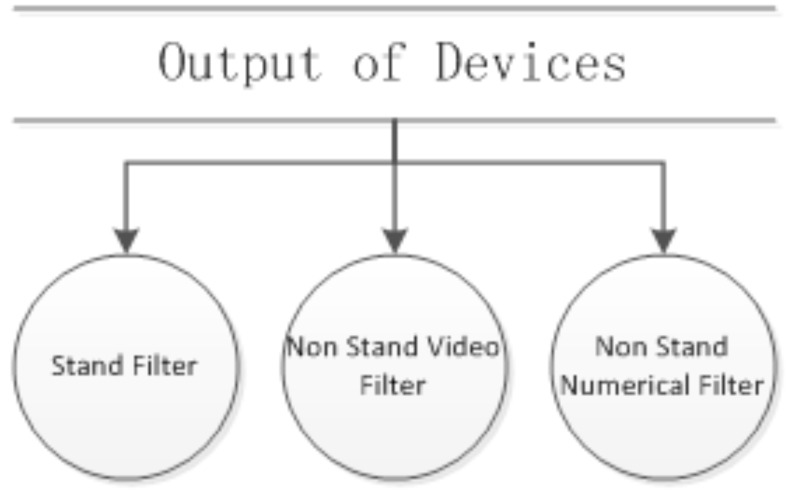
Structure map of the data filtering layer.

**Fig. (5):**
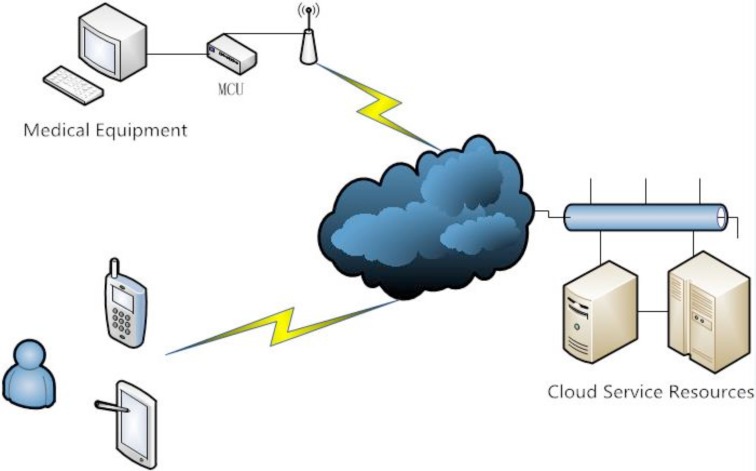
The schematic diagram of system extensions.

**Devices:**
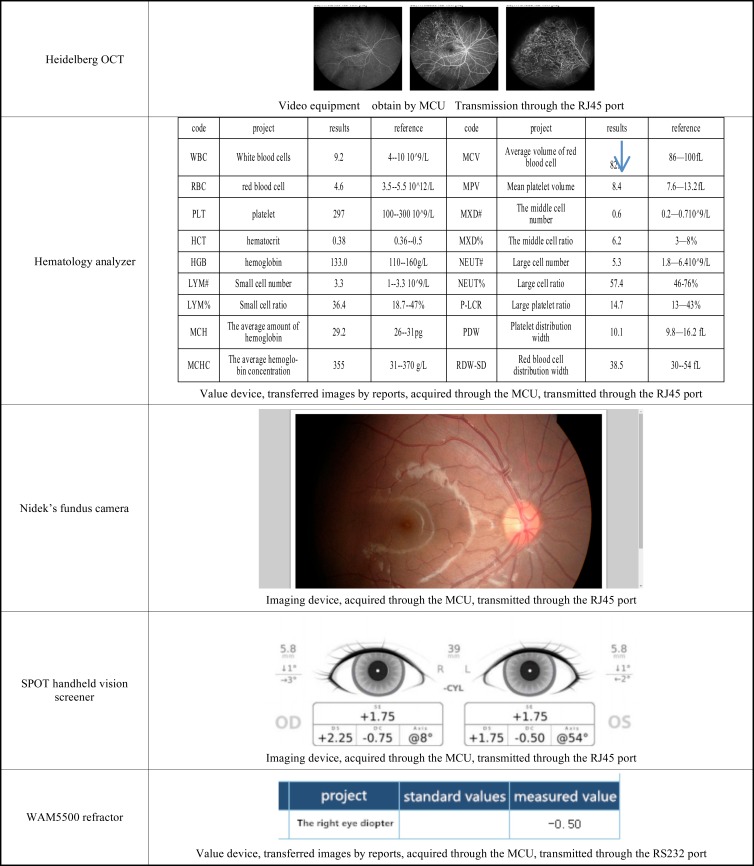
Below are the output examples of some devices:

## References

[1] Chowdhury B., D'Souza C., Sultana N. (2009). The use of emerging technology to improve the performance of health service delivery. Proc. IEEE Region 10 Conf. (TENCON-09).

[2] Kuhn K.A., Giuse D.A. (2001). From hospital information systems to health information systems. Problems, challenges, perspectives.. Methods Inf. Med..

[3] Beyer M., Kuhn K.A., Meiler C., Jablonski S., Lenz R. (2004). Towards a flexible, Proeess-oriented IT Architecture for an integrated healthcare network..

[4] Fung K.H., Low G.C. (2003). Design Notation for Dynamic Evolution in Com-ponent Based Distributed Systems. 7th IEEE Int. Enterp. Distribut. Object Comput. Conf..

[5] Grimes S.L. (2003). The future of clinical engineering: the challenge of change.. IEEE Eng. Med. Biol. Mag..

[6] Fung K.H., Low G., Ray P.K. (2004). Embracing dynamic evolu-tion in distributed systems.. IEEE Softw..

[7] Jammes F., Mensch A., Smit H. (2007). Service-oriented device communications using the devices profile for web services. 21^st^ Int. Conf. Adv. Inform. Networking Appl. Workshops.

[8] Barisic D., Krogmann M., Stromberg G., Schramm P. (2007). Making embedded software development more efficient with SOA. 21^st^ Int. Conf. Adv. Inform. Networking Appl. Workshops.

[9] Deb K., Pratap A., Agarwal S., Meyarivan T. (2002). A fast and elitist multiobjective ge-netic algorithm: NSGA-II.. IEEE Trans. Evol. Comput..

[10] Booehever S.S. (2004). HIS/R15/PACS integration:getting to the gold standard.. Radiol. Manage..

[11] Yan B., Huang G.W. (2009). Supply chain information transmission based on RFID and internet of things.

[12] Gkatehakis D., Tsiknakis M. (2002). Towardsan integrated electronie health reeord-current statusand challenges.

